# Intelligence Deficits in Chinese Patients with Brain Tumor: The Impact of Tumor Resection

**DOI:** 10.1155/2013/261236

**Published:** 2013-10-30

**Authors:** Chao Shen, Rong Xie, Xiaoyun Cao, Weimin Bao, Bojie Yang, Ying Mao, Chao Gao

**Affiliations:** ^1^Department of Neurosurgery, Huashan Hospital, Fudan University, Shanghai 200040, China; ^2^Institute of Neurology, Fudan University, Shanghai 200040, China

## Abstract

*Background*. Intelligence is much important for brain tumor patients after their operation, while the reports about surgical related intelligence deficits are not frequent. It is not only theoretically important but also meaningful for clinical practice. *Methods*. Wechsler Adult Intelligence Scale was employed to evaluate the intelligence of 103 patients with intracranial tumor and to compare the intelligence quotient (IQ), verbal IQ (VIQ), and performance IQ (PIQ) between the intracerebral and extracerebral subgroups. *Results*. Although preoperative intelligence deficits appeared in all subgroups, IQ, VIQ, and PIQ were not found to have any significant difference between the intracerebral and extracerebral subgroups, but with VIQ lower than PIQ in all the subgroups. An immediate postoperative follow-up demonstrated a decline of IQ and PIQ in the extracerebral subgroup, but an improvement of VIQ in the right intracerebral subgroup. Pituitary adenoma resection exerted no effect on intelligence. In addition, age, years of education, and tumor size were found to play important roles. *Conclusions*. Brain tumors will impair IQ, VIQ, and PIQ. The extracerebral tumor resection can deteriorate IQ and PIQ. However, right intracerebral tumor resection is beneficial to VIQ, and transsphenoidal pituitary adenoma resection performs no effect on intelligence.

## 1. Introduction

It is widely accepted that brain tumors are closely related to cognitive deficits. Cognitive function is increasingly regarded as an important prognosis index in patients with brain tumor [1]. Intelligence involves such domains of cognition as verbalization, memory, abstraction, planning, and execution function [2]. In 1980s, Warrington et al. [3] valued 656 patients with Wechsler Adult Intelligence Scale (WAIS), finding that unilateral cerebral lesions affected the intelligence. Petrucci et al. [4] and Taphoorn and Klein [1] reported that intracranial tumors caused cognitive impairments. Furthermore, pituitary adenomas were associated with cognitive impairments, even after successful surgical treatment, as reported by Tooze et al. [5] and Dorn et al. [6].

Because of the fact that survival life has been prolonged in patients with glioma, many researchers have dedicated themselves to the investigation of its cognitive effect and related adjuvant therapies. However, there is a dearth of literature in the comparative effect on intelligence between some benign and malignant brain tumors, for example, between benign meningioma and glioma. 

Surgical intervention, as a main invasive treatment, can change brain anatomical and functional integrity, causing a significant effect on postoperative cognitive status, which deserves as much attention as other confounding treatments as radiotherapy and chemotherapy [7–9]. However, the data on the immediate cognitive status following a brain tumor resection is limited.

Therefore, the current study took extracerebral tumor and pituitary adenoma into account and performed a postoperative immediate followup to investigate the surgery-related effect on patients' intelligence.

## 2. Methods

### 2.1. Participants

All inpatients were enrolled in the current study according to the following criteria: (1) aged between 16 and 70; (2) educated no less than elementary level; (3) diagnosed imageologically with a brain tumor in the supratentorial area or sellar region; (4) with a confirmed history of no brain surgery, psychosis, or mental disorder; (5) with an exclusion of a history of drug abuse or encephalopathy. Altogether, 103 inpatients treated in Huashan Hospital from October 2010 to October 2012 were enrolled, 57 women and 46 men (age range, 16–67 years; *M*_age_, 38.65; years of education, 6–20; *M*_edu_, 11.81). All the participants were right-handed; 7 of them refused to undergo a surgery, and 2 failed to receive the preoperative full assessment of WAIS for their incapability. A collection of 22 controls were acquired from the healthy staff and patients' families without any history of neuropsychological or physical illness. Informed consent was given to all the participants prior to the assessment.

### 2.2. Classifications (Table 1)

Based on tumor location, 103 subjects were divided into 3 subgroups (Classification I), 36 in one of the extracerebral tumors, 54 in one of the intracerebral tumors and 13 in one of the pituitary adenomas, which was excluded from the investigation for their medical incomparability. The first two subgroups and the 22 healthy controls were subjected to statistical comparison, which showed no significant difference in their age (*F* = 1.792, *P* = .171), their years of education (*F* = 1.420, *P* = .246), and their tumor size (*P* = .177).

Based on tumor lateralization, 83 subjects were divided into 2 subgroups (Classification II), 45 in one of the left hemisphere tumors and 38 in one of the right hemisphere tumors, with an exclusion of 13 with pituitary adenoma and 7 with bilaterally involved brain tumor. The two subgroups of those included and the 22 healthy controls were subjected to statistical analysis, which showed no significant difference in their age (*F* = .824, *P* = .441), their years of education (*F* = 1.503, *P* = .227), and their tumor size (*P* = .195).

For further detailed analysis, 83 were classified into 4 groups against the specific location and lateralization of brain tumor (Classification III): 15 in the left-extra subgroup, 15 in the right-extra subgroup, 30 in the left-intra subgroup and 23 in the right-intra subgroup. 

Postsurgically, 56 patients who had undergone craniotomy and 11 subjects who had received transsphenoidal pituitary adenoma resection, respectively, completed a full assessment.

### 2.3. Intelligence Assessments

WAIS was performed by two colleagues trained by a neuropsychologist. If the patients' physical condition permitted, a seven-subtest short form of the WAIS, that is, information, similarities, picture completion, vocabulary, digit span test, picture assignment, and block design, was used to evaluate the patients' intelligence at admission and discharge, respectively, for the validity and clinical utility of the seven-subtest WAIS short form in patients with brain tumor are high [10]. To minimize the memory effect of duplicated assessment, the answers to the questions in WAIS and the notification of the postoperative reassessment were absolutely concealed from the subjects. 

### 2.4. Statistical Analysis

Analysis of variance (ANOVA) was conducted to determine statistical differences among all the subgroups, and univariate analysis was followed by post hoc comparisons with LSD correction. Partial correlation and paired-sample *t*-test were used to analyze the effect of age, years of education, tumor size, and surgery. All statistical analysis was performed by SPSS version 11.5 for Windows.

## 3. Results

### 3.1. Preoperative IQ of Subgroups

**Figure 1:**
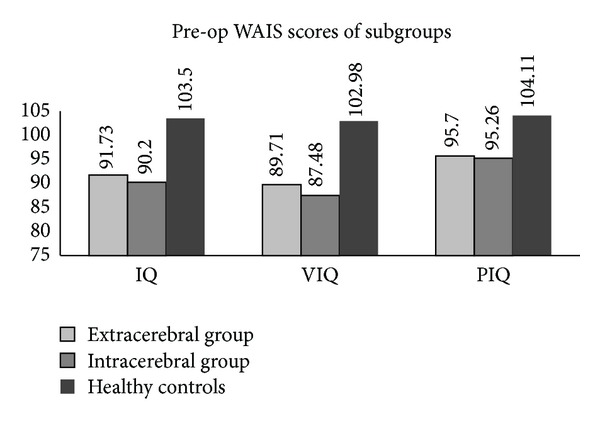
Preoperative IQ, VIQ, and PIQ of both the intracerebral and the extracerebral subgroups were lower than those of the healthy controls (*P* < .05). However, no significant difference was demonstrated between these two tumor subgroups.

**Figure 2:**
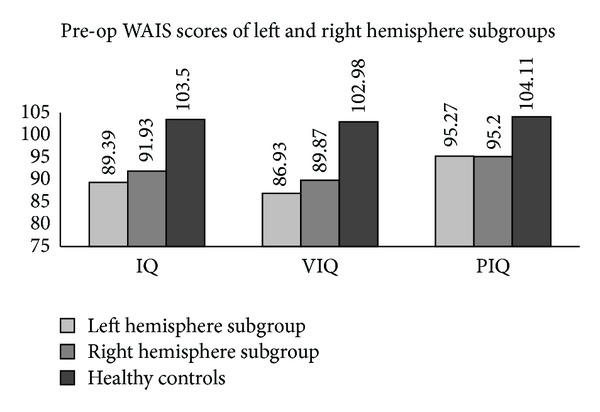
Preoperative IQ, VIQ, and PIQ of both left and right hemisphere subgroups were lower than those of the healthy controls (*P* < .05). No significant difference was found between the two tumor subgroups.

The preoperative IQ, VIQ, and PIQ of all the subjects were found to be lower than those of the healthy controls. However, they showed no significant differences between the intracerebral and extracerebral subgroups of Classification I as well as between the left and right hemisphere subgroups of Classification II (Figures 1 and 2).

It was found that VIQ was significantly lower than PIQ in those with brain tumor (*P* = .001), whether it was right-sided or left-sided, intracerebral or extracerebral.

**Figure 3:**
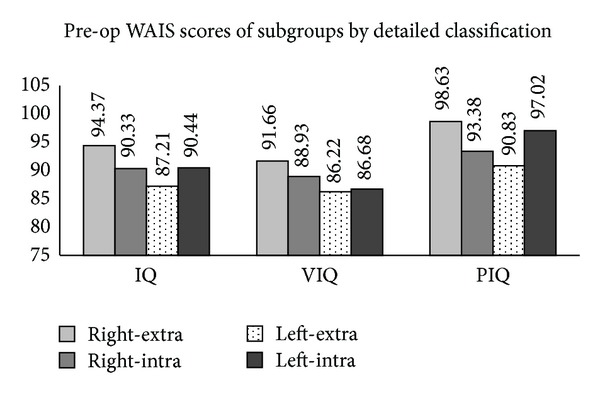
From pre-op WAIS scores of subgroups by detailed classifications, no significant difference was observed in IQ (*F* = .654, *P* = .583), VIQ (*F* = .520, *P* = .670), and PIQ (*F* = 1.184, *P* = .321) among subgroups.

The analysis based on Classification III showed that there was no significant difference among the subgroups in terms of IQ, VIQ, and PIQ (Figure 3).

### 3.2. Immediate Postoperative Followup

**Figure 4:**
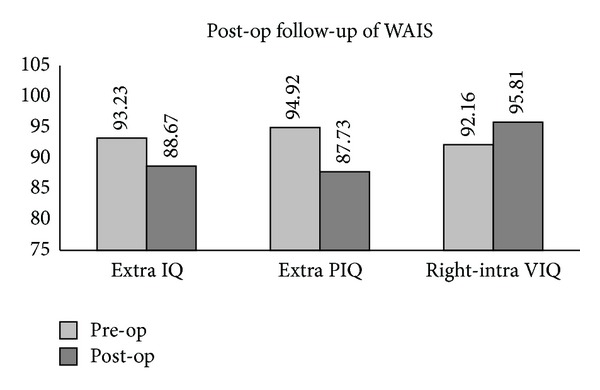
Significant decline of the IQ (*P* = .024) and PIQ (*P* = .004) was found in the extracerebral subgroup after surgery, and the VIQ of the right intracerebral subgroup significantly improved (*P* = .009).

Postoperatively, IQ (*P* = .024) and PIQ (*P* = .004) of the extracerebral subgroup declined significantly, as indicated by Classification I. Nevertheless, the results of Classification III showed that VIQ (*P* = .009) of the right intracerebral subgroup improved significantly, which was not observed in the left intracerebral one (Figure 4).

### 3.3. Partial Correlation

**Figure 5:**
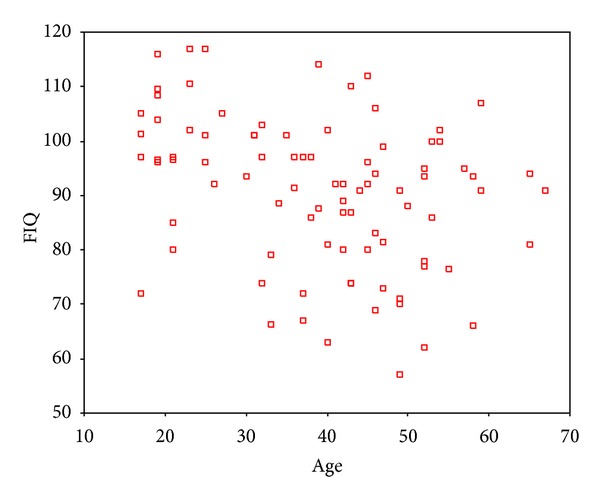
Partial correlation analysis showed a negative relation between age and IQ. Correlation coefficient was −0.287 (*P* = .008).

**Figure 6:**
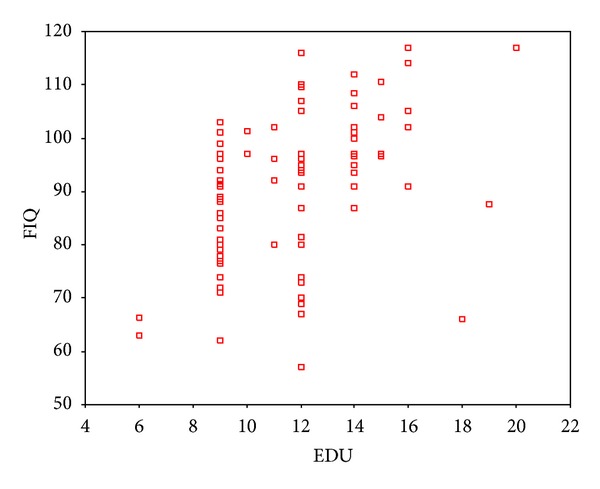
Partial correlation analysis showed a positive relation between years of education and IQ. Correlation coefficient was 0.448 (*P* = .000).

As indicated by partial correlation analysis, IQ showed a negative correlation with age, but a positive one with the years of education (Figures 5 and 6). 

**Figure 7:**
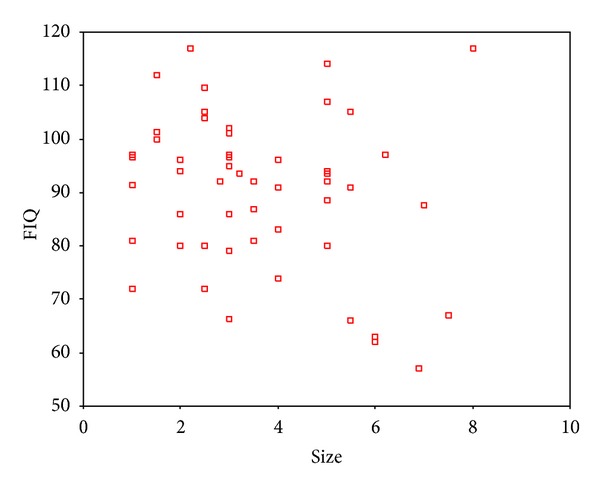
Partial correlation analysis showed a negative relation between tumor size and IQ in the intracerebral subgroup. Correlation coefficient was −0.383 (*P* = .005).

**Figure 8:**
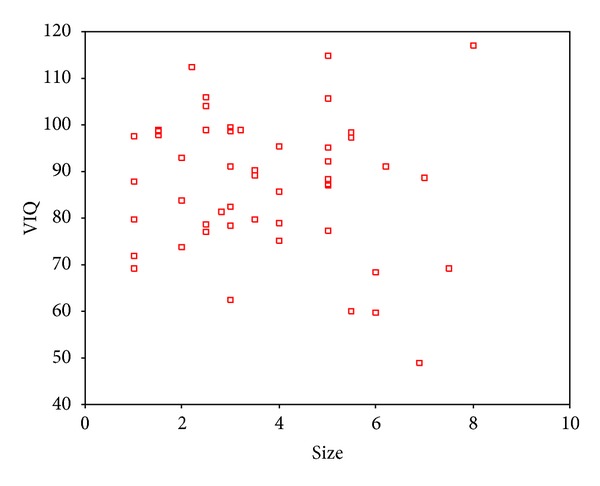
Partial correlation analysis showed a negative relation between tumor size and VIQ in the intracerebral subgroup. Correlation coefficient was −0.280 (*P* = .047).

**Figure 9:**
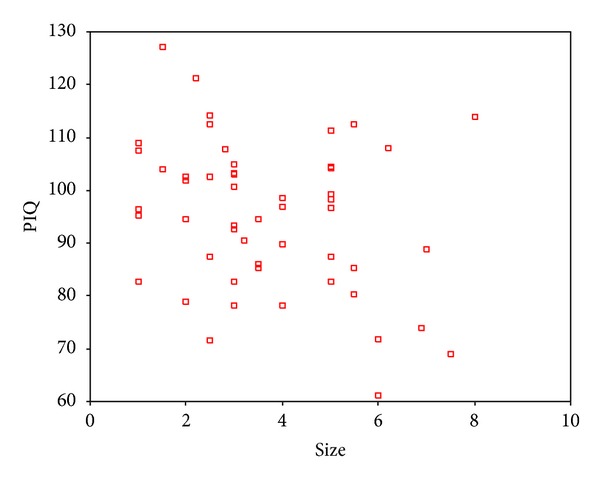
Partial correlation analysis showed negative relation between tumor size and PIQ in the intracerebral tumor subgroup. Correlation coefficient was −0.432 (*P* = .002).

The tumor size was found to be negatively correlated with IQ, VIQ, and PIQ, respectively, exclusively in the intracerebral subgroup (Figures 7, 8, and 9).

### 3.4. Three-Month Followup

**Figure 10:**
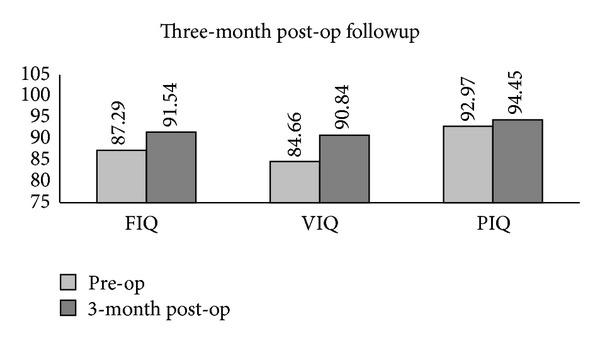
Improved VIQ (*P* = .001) was found in 14 patients three months after surgery.

The full assessments of the 14 subjects, obtained at the followup of three months, indicated an improved VIQ (*P* = .001) when compared with that prior to their surgeries (Figure 10). 

## 4. Discussions

### 4.1. Tumor Malignancy, Location, and Lateralization

Many cognitive investigations have dealt with low grade glioma, because patients with glioma live relatively a long survival life after surgery and adjuvant therapy, with an agreement on the negative effects on their cognition [1, 11–13]. Patients with high-grade neoplasms have been proved to do more poorly in the neuropsychological test than those with low-grade tumors [14]. Scheibel's research, however, showed that malignancy grade exerted no effect on intelligence [15]. The current study proved that intelligence deficits occurred in patients with brain tumor, but did not show that tumor malignancy correlated with intelligence deficits. As indicated, no siginificant difference was found in IQ, VIQ, and PIQ between the patients with benign tumors and those with malignant ones before the operation.

It was previously reported that patients with brain tumors of different localization and lateralization tended to develop the neuropsychological patterns of dysfunction in various ways [16]. Warrington reported that VIQ was impaired in all patients with left-hemisphere lesion and that right-hemisphere lesion led to a dysfunction on the abstract concept, visuospatial ability, and graph recognition, especially on those with right parietal involvement [3]. Luria, Hong Wang and Gong, Glanzer, and Bryden also indicated that the left hemisphere controlled VIQ and verbal memory while the right reflected PIQ and nonverbal abstract memory [2, 17–19]. In the current study, no significant difference was found between the left and right subgroups in terms of VIQ or PIQ, except that VIQ is significantly lower than PIQ before surgery. And also, the right-sided tumor induced lower scores in verbal tests. We were more inclined to accept the view that the cognitive deficits due to brain tumor were different from traumas or cerebrovascular diseases; therefore, they could not be simply restricted to any single domain. Even though some deficits were related to tumor location, the patients tended to present global cognitive deficits. Furthermore, VIQ was found to be much more vulnerable than PIQ in the Chinese population, which could be explained by the uniqueness and complexity of Chinese characters, for their remembering and processing can involve both verbal and nonverbal domains. Japanese investigators even reported that it mainly depended on the right hemisphere to distinguish kanji [20, 21].

### 4.2. Surgical Effect

Yoshii demonstrated that preoperative and postoperative cognitive functions were normal in patients with glioma, except in those with left malignant glioma, and that the cognitive function was not improved in those with left-sided meningioma [22], where modified MMS for assessment is commonly applied to dementia; therefore, it failed to detect subtle cognitive deficits. However, Tucha showed no change of memory, visuoconstructive abilities, or executive function in patients with frontal meningioma resection [23], and an amazing improvement of functioning in various domains of cognition was displayed in the elder patients with falx cerebri meningioma [24].

The current study partially agreed on Yoshihiko's view, but disagreed on Tucha's, because it was not the intracerebral tumor resection but the extracerebral tumor that made IQ and PIQ even worse, regardless of lateralization. Lezak reported that diffused cerebral dysfunction, which cannot be accurately located, would decrease PIQ scores [25]. We presumed that it could be a decrease in the whole brain function rather than the local compression which could impair PIQ. It can be that the local brain tissues compressed by an extracerebral tumor may still maintain the local anatomical and functional integrity before surgery and that local brain damages are always kept lowest during an extracerebral tumor resection. After decompression due to tumor resection, brain remodeling and shifting made a rapid and obvious decline in the whole brain function, thus affecting multiple cognitive domains and impairing PIQ performance. 

Furthermore, only right intracerebral tumor resection was found to improve VIQ, which did not occur in the left hemisphere or extracerebral subgroup. Surgery on glioma involves the resection of both the tumor and suspected brain tissues, which means that the operation damages the local brain function thoroughly. However, the results did not show a postoperative significant decline in intelligence, suggesting that the local cortex might not play a vital role in intelligence and that some unknown compensation mechanisms might work in other areas, which merits further studies. 

It is worth mentioning that lateralization is still postoperatively important for cognitive recovery and that the intact function of the left-sided hemisphere is essential for VIQ rehabilitation. In addition, the transsphenoidal approach is suggested to be safe on patients with pituitary adenoma in terms of intelligent performance. 

### 4.3. Other Impact Factors

It has been reported that age is negatively correlated with cognitive status, while years of education is positively correlated, which is in agreement with our findings. Tucha indicated that the cognition of patients with brain tumors was influenced by the size of the tumor [26]. From our statistical results, however, this negative correlation was only observed in the intracerebral subgroup, which suggests that the brain tissues are more capable of compensation for compression due to an extracerebral tumor. For example, one patient with a huge meningioma of 11 cm in diameter suffered from a mild neurological and cognitive deficits exclusively. But this is not the case for an intracerebral tumor. In addition to shifting effect, it can cause direct damages to the cortex. With an increase in the size of tumor, brain edema and intracranial hypertension accompanied by severe clinical symptoms are common. Therefore, tumor size exerted a significant effect on intelligence in the intracerebral subgroup.

### 4.4. Three-Month Postoperative Followup

Fortunately, the current study received 14 follow-up visits three months after craniotomy for the full assessment of WAIS. From the investigation, a significant improvement was found in VIQ, but in PIQ no change was observed when compared with those before surgery, respectively. The rest of the subjects were followed up over the phone, with approximately 73% of them showing normal language function and self-care ability. Though the physical follow-up rate was low, we believe that a recovery from preoperative VIQ impairment could still be anticipated. 

## 5. Conclusions

In conclusion, brain tumor can definitely induce IQ, VIQ, and PIQ impairments before surgery, while no significant difference was found between the intracerebral and extracerebral subgroups in the current study. Our results indicated that VIQ was lower than PIQ in patients with brain tumor; that lateralization did not affect preoperative intelligence; that extracerebral tumor resection deteriorated IQ and PIQ while the right intracerebral tumor resection improved VIQ; that tumor size was negatively correlated with IQ, VIQ, and PIQ in patients with an intracerebral tumor; that pituitary adenoma and its transsphenoidal approach failed to impair intelligence; and that VIQ improvement could be anticipated in those who were likely to recover physically, all of which can be treated as preoperative information for the consulted surgeons.

## Figures and Tables

**Table 1:** Demographic characteristics of all subgroups (M ± SD).

Subgroup	*N*	Age	Years of education
		(M ± SD)	(M ± SD)
Healthy controls	22	38.86 ± 12.12	12.50 ± 3.42
Pituitary adenoma subgroup	13	34.31 ± 10.77	13.85 ± 3.58
Extracerebral subgroup	36	41.31 ± 11.83	11.17 ± 2.30
Intracerebral subgroup	54	37.93 ± 13.73	11.74 ± 3.09
Left hemisphere subgroup	45	38.91 ± 12.08	12.00 ± 3.20
Right hemisphere subgroup	38	38.74 ± 14.21	11.00 ± 2.30
Craniotomy	56	37.27 ± 12.59	11.31 ± 2.71
Transsphenoidal approach	11	34.91 ± 11.37	13.18 ± 3.43
